# Novel strategies in Parkinson’s disease treatment: a review

**DOI:** 10.3389/fnmol.2024.1431079

**Published:** 2024-08-09

**Authors:** Charles L. Mitchell, Dmitry Kurouski

**Affiliations:** ^1^Interdisciplinary Program in Genetics and Genomics, Texas A&M University, College Station, TX, United States; ^2^Department of Biochemistry and Biophysics, Texas A&M University, College Station, TX, United States

**Keywords:** cell therapy, Parkinson’s disease, amyloids, macrophages, natural kill cell

## Abstract

An unprecedented extension of life expectancy observed during the past century drastically increased the number of patients diagnosed with Parkinson’s diseases (PD) worldwide. Estimated costs of PD alone reached $52 billion per year, making effective neuroprotective treatments an urgent and unmet need. Current treatments of both AD and PD focus on mitigating the symptoms associated with these pathologies and are not neuroprotective. In this review, we discuss the most advanced therapeutic strategies that can be used to treat PD. We also critically review the shift of the therapeutic paradigm from a small molecule-based inhibition of protein aggregation to the utilization of natural degradation pathways and immune cells that are capable of degrading toxic amyloid deposits in the brain of PD patients.

## Introduction

**Figure 1:**
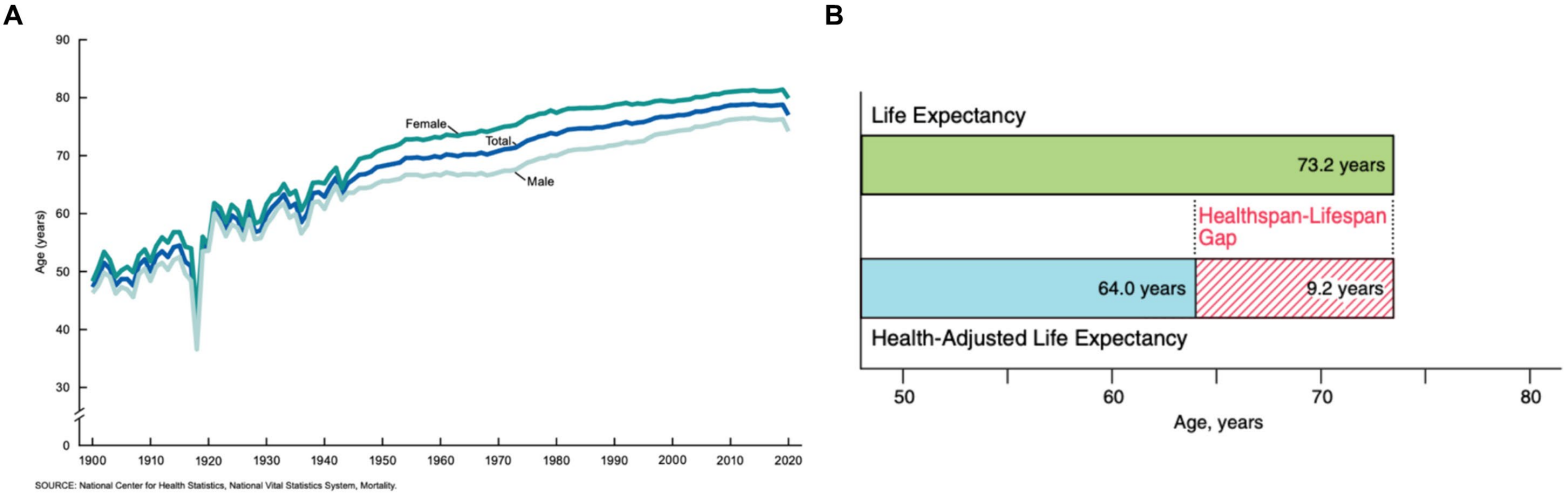
**(A)** The average life expectancy of U.S. citizens as collected from 1900 to 2020 (Arias and Xu, 2022). **(B)** The average predicted healthspan-lifespan gap of adults in the U.S. in 2020 (Garmany et al., 2021).

At the turn of the twentieth century, our civilization faced an unprecedented boom in human lifespan. This phenomenon was primarily attributed to a substantial progress in medicinal practices, discovery of antibiotics, and major improvement of sanitary practices. In the U.S. in 1900, an average life expectancy was under 50 years old, while in the mid-80s the average lifespan increased to 79 years (Figure 1A; Arias and Xu, 2022). Continuous growth of the lifespan suggests that the number of people over the age of 60 will double in the next three decades reaching 2.1 billion by 2050 (Bejot and Yaffe, 2019). This significant increase in life expectancy poses a threat to public health, as health expectancy in the population has not increased at a proportional rate (Crimmins, 2015).

Health expectancy, also known as healthspan, is the average expected number of disease-free years for a given population. In the U.S. in 2020, the average healthspan was only 64.0 years, while the lifespan was 73.2 years (Garmany et al., 2021) (Figure 1B). Senior individuals suffer from a large spectrum of pathologies including Parkinson’s disease (PD), Alzheimer’s disease (AD), Lewy body dementia (LBD), and amyotrophic lateral sclerosis (ALS). There are approximately 60,000 cases of only PD diagnosed annually in the U.S. alone, with a medical market estimate worth at least $52 billion (Chopade et al., 2023). Furthermore, PD is the quickest increasing neurological disorder, and the market is expected to proportionally increase as an estimated 12 million patients will be diagnosed worldwide by 2040 (Chen, 2010). Although the exact cause of such pathologies remain unclear, a growing body of evidence suggests that an onset and spread of neurodegeneration are triggered by the abrupt aggregation of misfolded proteins (Beitz, 2014).

An eukaryotic cell contains thousands of different proteins, with some estimates of over 500,000 proteins encoded by 20,000 protein-encoding genes (Pray, 2008). The number of proteins produced is much higher than the number of genes that encodes them due to alternative splicing. Alternative splicing is the process in which a single protein-encoding gene can produce multiple different distinct protein products depending on how its exons are spliced together. Experiencing high levels of proteins results in the cell maintaining protein homeostasis, or proteostasis, via balancing the systems for protein synthesis, correct protein folding, post-translational processing, cellular localization, and the protein degradation systems: the ubiquitin-proteasome system (UPS) and the autophagy-lysozyme pathway (Balch et al., 2008; Hommen et al., 2022). Due to the brain’s absence of the traditional lymphatic vasculature, it relies on the glymphatic system to remove the waste products of the proteostasis network (Wardlaw et al., 2020; Beschorner and Nedergaard, 2024). Internal and external cellular stresses impact the functionality of the proteolytic mechanisms that maintain proteostasis, including misfolded\ proteins, genetic mutations, viral and bacterial infections, physical and environmental stresses, lifestyle choices, pharmacological stresses, and aging. An increase in the concentration of unfolded proteins in the cell triggers several molecular mechanisms, including unfolded protein response (UPR), that aim to restore normal cell functioning (Hohn et al., 2020; Hommen et al., 2022). These mechanisms involve an arrest of transcription and translation, as well as an increase in the concentration of chaperons, molecular systems that facilitate protein folding. The impact of a malfunctioning proteostasis network is exaggerated in nondividing, long-lived cells like neurons. One may expect that these and other factors trigger devastating neurodegenerative diseases such as discussed above AD, PD, ALS, and dementia (Hohn et al., 2020).

**Figure 2:**
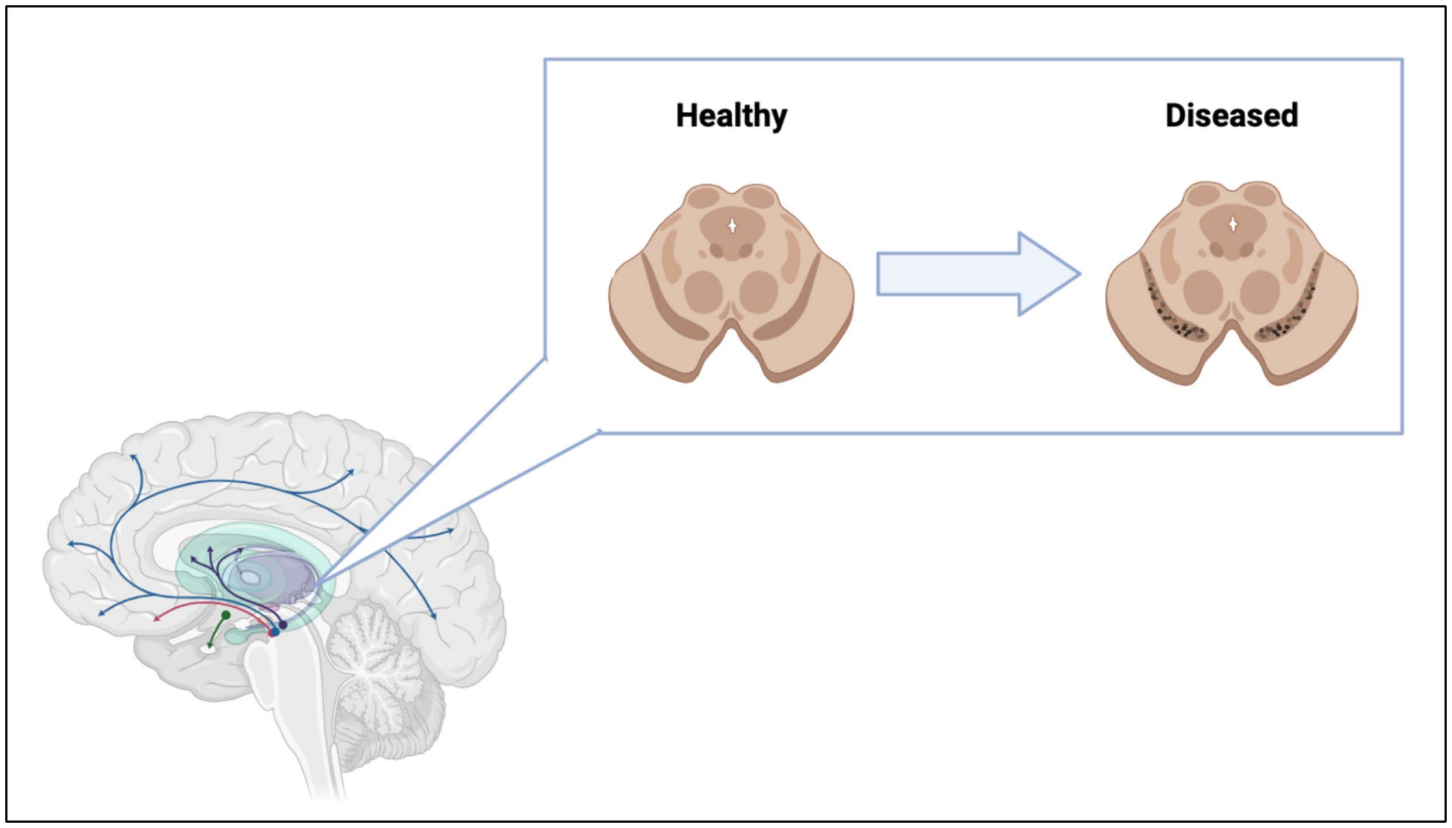
Loss of dopaminergic neurons in the substantia nigra lead to interruption of dopamine pathways in the brain. Figure was created using BioRender.com.

Microscopic examination of brains of patients diagnosed with these pathologies revealed the presence of intra and extracellular formations. These inclusions are dominated by protein aggregates and fragments of cell membranes (Boland et al., 2018; Chung et al., 2018). Specifically, PD is clinically characterized by Lewy bodies, formations first detected in substantia nigra pars compacta (SNc). This brain region is dominated by dopaminergic (DA) neurons. It was hypothesized that DA neurons are more sensitive to disease because of their innate demand for large amounts of energy required to maintain neuronal signaling via the high number of axonal arborizations (Wong et al., 2019). Irreversible degeneration of DA neurons results in the interruption of dopamine transport in the motor loop pathways of the basal ganglia (Kingsbury et al., 2010) (Figure 2). The loss of dopamine pathways results in multiple physical symptoms of PD such as bradykinesia, postural tremors and rest tremors, muscle rigidity, and postural instability (Varadi, 2020), as well as the psychiatric symptoms of PD such as depression, mood swings, anxiety, psychosis, and apathy (Han et al., 2018). The majority of PD patients also develop at least one form of autonomic dysfunction, including cardiovascular, genitourinary, thermoregulatory, and gastrointestinal (Han et al., 2022). Often, gastrointestinal dysfunction and pathology have preceded mental decline by years or decades (Tan et al., 2023), although not seen in all patients. Gastrointestinal symptoms worsen as PD progresses, with approximately 60–80% of PD patients experiencing gastrointestinal issues at some point before or during prognosis (Tan et al., 2023). Patients can have upper gastrointestinal symptoms, like sialorrhea (Warnecke et al., 2022), dysphagia, oropharyngeal dysfunction, aspiration pneumonia, and gastroparesis, coupled with or independent of lower gastrointestinal symptoms, like constipation, irregular bowel movements, and straining while defecating (Skjaerbaek et al., 2021; Warnecke et al., 2022). Interestingly, James Parkinson, the first person to give a formal, detailed description of the disease, suggested that PD may have its origins in the gastrointestinal tract, stating, “a disordered state of the stomach and bowels may induce a morbid action in a part of the medullar spinalis” (Pfeiffer, 2018; Tan et al., 2022). Research exploring the gut-brain axis and how it pertains to PD has long been understudied but has increased within the last 20 years.

The progressive degeneration of DA neurons is linked to the aggregation of *α*-synuclein (*α*-syn), a small 140 amino acid long intrinsically disordered protein (IDP) encoded by the *SNCA* gene located on chromosome 4 (Stefanis, 2012; Oczkowska et al., 2013). *α*-Syn is primarily localized in the presynaptic terminal of DA neurons. However, recent studies demonstrated that *α*-syn was also present in mitochondria (Li et al., 2007), endoplasmic reticulum (Hoozemans et al., 2007), Golgi apparatus (Gosavi et al., 2002), and nuclei of neurons (Maroteaux et al., 1988). Although its exact physiological function remains unclear (Bernal-Conde et al., 2019), *α*-syn is involved in SNARE transporters (Burre et al., 2013). It also plays an important role in sensing and stabilizing curved membranes (Shen et al., 2012). Native *α*-syn has strong binding propensity to negatively charged phospholipids. This results in the major transformations of the protein secondary structure from unordered to *α*-helical (Burre et al., 2013; George and Yang, 2013; Li et al., 2022). In its membrane bound conformation, the non-amyloid component (NAC) domain of *α*-syn can toggle between being buried in the membrane and being exposed into the cytosol (Fusco et al., 2014; Hijaz and Volpicelli-Daley, 2020). When exposed into the cytosol, the hydrophobic NAC domain triggers the aggregation of monomeric *α*-syn into highly toxic oligomers and fibrils (Waxman et al., 2009; Du et al., 2020; Hijaz and Volpicelli-Daley, 2020). These protein aggregates primarily have cross-ß-sheet secondary structure (Rodriguez et al., 2015; Tuttle et al., 2016; Meade et al., 2019; Guerrero-Ferreira et al., 2020). *α*-Syn oligomers and fibrils leads to the death of neurons and severe neurodegeneration (Du et al., 2020; Hijaz and Volpicelli-Daley, 2020; Dou et al., 2023) the onset of PD (Calabresi et al., 2023).

**Figure 3:**
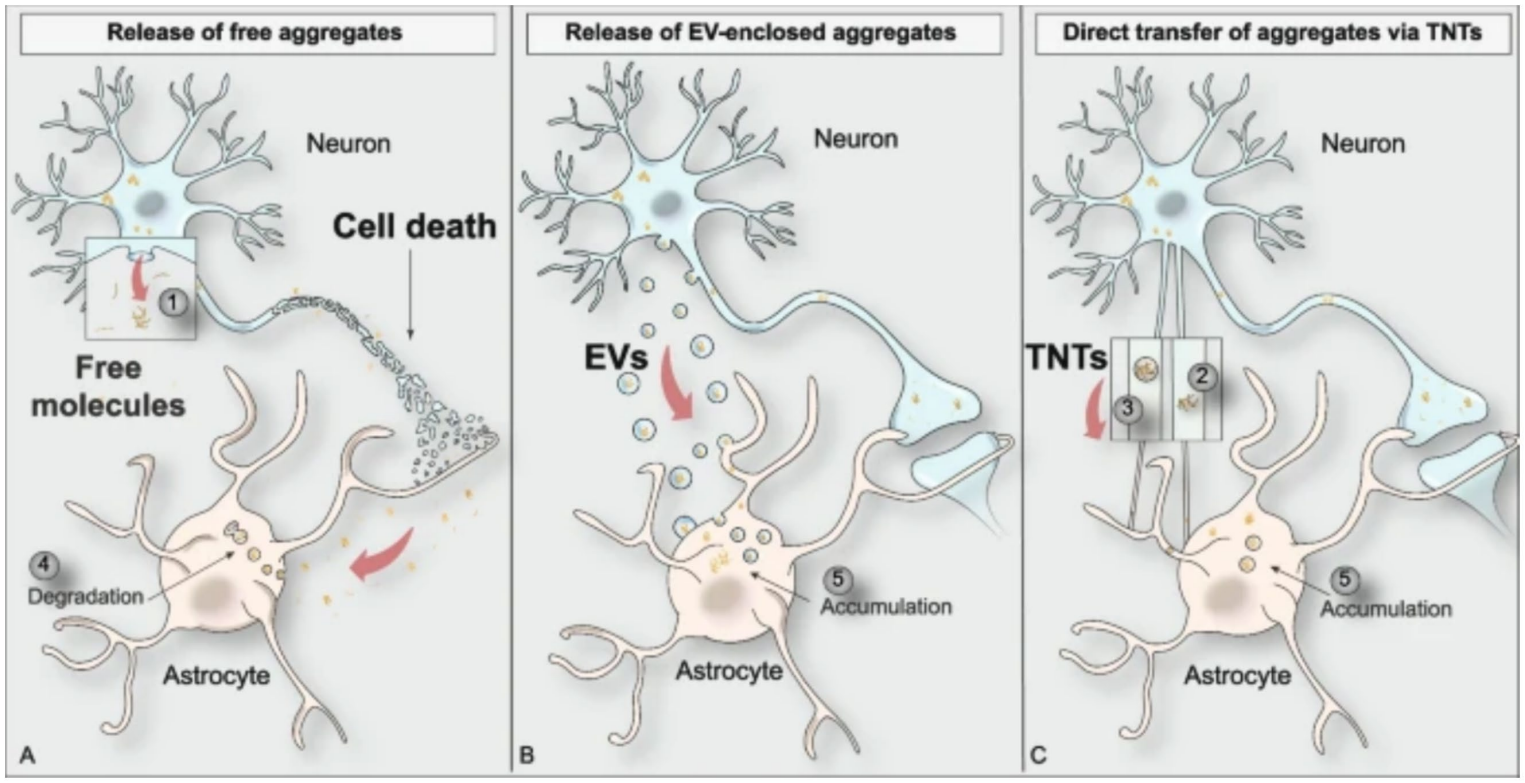
Visual depiction of the three main modes of protein aggregate spreading between cell types in the brain (Giusti et al., 2024).

Throughout the progression of PD pathology, *α*-syn oligomers and fibrils are transferred between diseased and healthy cells leading to the spread of PD. Tracking of PD progression gives rise to the diagnosis of Braak’s staging in PD patients used in the research and clinical settings. There are three main mechanisms of *α*-syn spread between cells in the brain: release of free aggregates as a result of cellular death, release of extracellular vesicles containing aggregates, and direct transfer of aggregates via intercellular tunneling nanotubes (TNTs) (Figure 3) (Baba et al., 1998; Arai et al., 2001; Goedert et al., 2013; Kim et al., 2014; Lim and Yue, 2015; Marotta et al., 2021; Giusti et al., 2024).

To better understand the molecular, biochemical, and anatomical aspects of PD, scientists in the field use a variety of disease models. Models used in PD research fall into two categories: organismal and cellular models. Organismal models allow for studying the effects of PD on the organism as a whole, while cellular models allow for studying the effects of PD on a specific cellular and biochemical level. The most widely used organism is the rat model, followed by mice and non-human primates. Rat models were used in 48% of published papers, followed by 37% of papers using mice models, and only 10% utilizing non-human primates (Konnova and Swanberg, 2018). There have been attempts at creating a swine model of PD due to the similar brain anatomy between pigs and humans. However, these genetically modified pig models have not proven successful, with PARK2 knockout only generated in fibroblasts, DJ-1 knockouts resulting in postnatal lethality, and PINK1 knockouts not developing PD symptoms (Holm et al., 2016).

The models used in PD research can be induced via two pathways: neurotoxin and genetic models (Pingale and Gupta, 2020). Each model induction type offers valuable insights into certain aspect of PD pathology, the biochemical environment of PD, and propagation of protein aggregates. However, in the field of protein aggregate clearance, the neurotoxin induced models are not used due to their lack of the *α*-syn aggregates. Neurotoxin induced models work by mimicking the loss of dopaminergic neurons in the midbrain by killing the neurons, not by inducing and propagation of protein aggregates (Calabresi et al., 2023).

## Amyloid clearance

**Figure 4:**
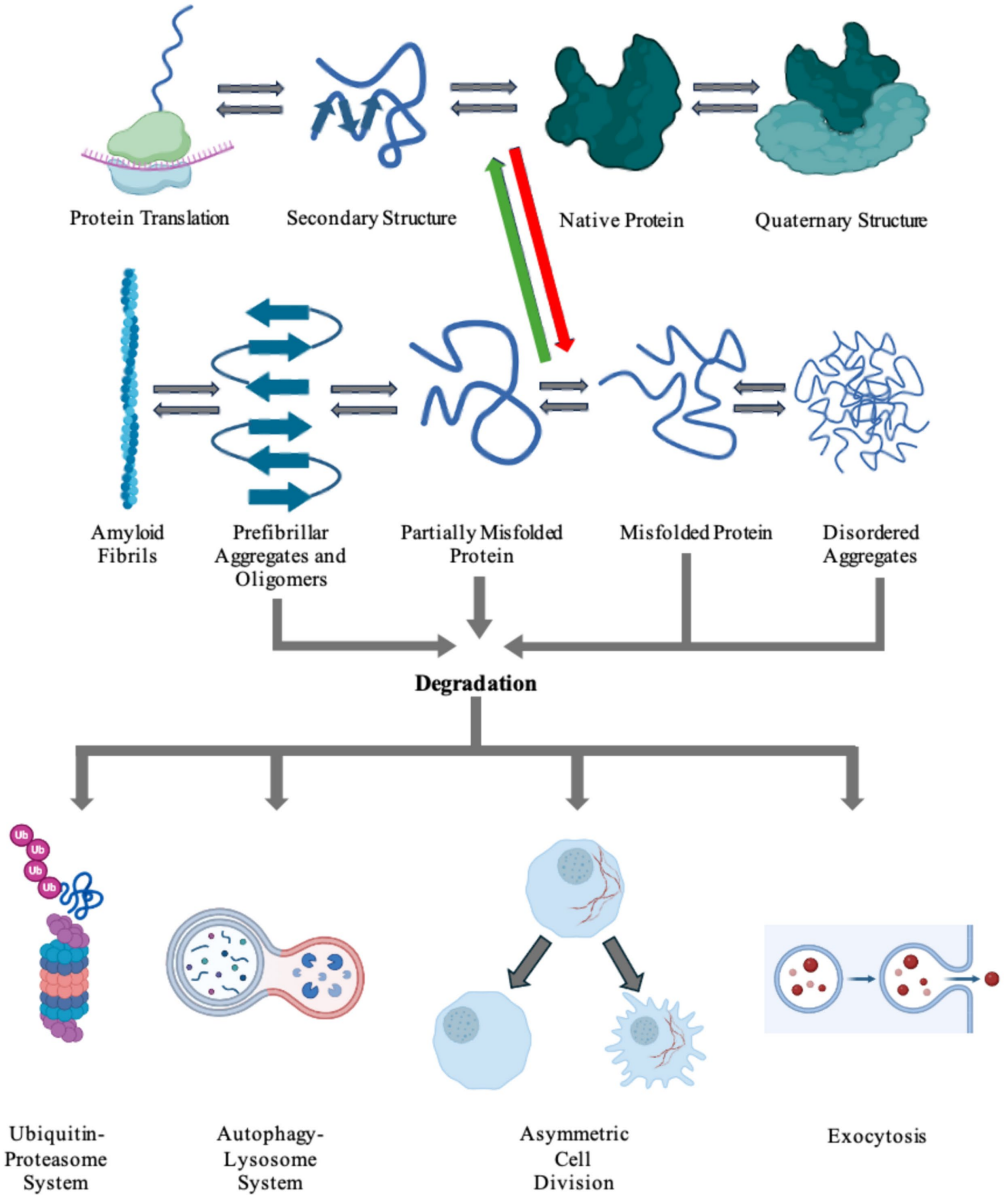
Cells harbor a complex network of biochemical processes responsible for the production and maintenance of essential proteins. The proteins are first transcribed from DNA to RNA, then translated into its inherent primary amino acid sequence at the ribosome. The produced amino acid chain is then partially folded into its secondary structure. The protein can then continue folding into its native tertiary structure with the help of molecular chaperones. If the protein colocalizes with other proteins, it can go on to form functional quaternary structures. Due to a number of factors, including genetic mutations and environmental stresses, the protein can misfold (red arrow). The cell is able to recognize the newly misfolded protein and can try to refold the protein back to its native form (green arrow) or label it for degradation. The protein can also propagate its misfolded conformation by forming either ordered or unordered aggregates. Disordered aggregates and other aggregates can be detected and fated for destruction via any of the degradation pathways: the ubiquitin-proteosome system, the autophagy-lysosome system, asymmetric cell division, or exocytosis. Highly ordered aggregates, amyloid fibrils, cannot be degraded by the cell and causes pathology in a number of diseases. Figure was created using BioRender.com.

Considering failure of numerous attempts to inhibit protein aggregation using small molecules, several research groups have been exploring alternative therapeutic concepts. These new treatment paradigms focus on large molecular complexes and immune cells. Eukaryotic cells have innate pathways for clearing misfolded, malfunctioning, aged, or aggregated proteins (Wen et al., 2023). These include refolding proteins into their normal physiological conformations, degrading proteins utilizing the ubiquitin-proteasome or autophagy-lysosome systems, asymmetric cell division (ACD), or exocytosis of aggregated protein via the extrusion of exosomes or exophers (Tyedmers et al., 2010; Dikic, 2017; Melentijevic et al., 2017; Mogk et al., 2018; Pohl and Dikic, 2019; Nicolas-Avila et al., 2020; Wen et al., 2023). Figure 4 shows the processes in which cells produce a nascent protein and folds the amino acid chain into a functional protein. However, there can be a disturbance in this pathway that causes the protein to misfold and aggregate. Cells then utilize internal mechanisms to fold or degrade misfolded proteins (Figure 4). Furthermore, immune cells, including natural killer (NK) cells, microglia, and astrocytes can be used to uptake and degrade protein aggregates in the brain (Menees and Lee, 2022; Ozoran and Srinivasan, 2023).

### Utilizing degradation machinery

The clearance of aggregated proteins ultimately leads to alleviation of classical clinical symptoms of disease. Lee and co-workers found that aryloxy propanolamine induces chemical dissociation and clearance of aggregated amyloid-ß (Aß) (Lee et al., 2022), the pathologic protein linked to AD. This compound dissociated the ß-sheet-rich aggregations of Aß, and upon oral administration reduced the amyloid burden in the brain of the mice tested. It was also shown that aryloxy propanolamine attenuated major pathological symptoms of AD including tauopathy, neuroinflammation, and synaptic protein loss (Lee et al., 2022).

Recently reported results by Saha and co-workers showed that ATPase valosin-containing protein (VCP) could be used to disaggregate Tau fibrils (Saha et al., 2023). Disaggregation in higher eukaryotes, like mammals, is traditionally associated with the heat shock protein 70 kDa (Hsp70) chaperone machinery (Shorter, 2011; Wentink et al., 2020; Saha et al., 2023). Several research groups demonstrated that human Hsp70-Hsp40-Hsp110 system has the intrinsic ability to dissociate Tau and *α*-syn fibrils *in vitro* (Gao et al., 2015; Nachman et al., 2020; Schneider et al., 2021), but is not as efficient *in vivo*. Human VCP exerts an ATP-dependent pathway responsible for unfolding proteins via the ubiquitin-proteasome system. When mutations in the VCP protein were introduced, accumulation of Tau aggregates were observed, as well as perturbed autophagic function (Tresse et al., 2010; Saha et al., 2023). Similar to VCP, other important members of the ubiquitin-proteasome system attracted an interest of the scientific community as a potential therapeutic for PD, AD, and other neurodegenerative diseases.

It was recently demonstrated that protein aggregates could be cleared by targeted protein degradation (TPD). Emerging technologies that fall under the umbrella of TPD include PROteolysis TArgeting Chimeras (PROTAC), Lysosome-Targeting Chimera (LYTAC), Antibody-based PROTAC (AbTAC), and molecular glue (Zhao et al., 2022). The idea of TPD was first introduced in 1999, but the first PROTAC molecule was not discovered for two more years in 2001. The TPD field stalled until 2019 when two PROTAC molecules were approved for clinical trials, then LYTAC was introduced in 2020, with AbTAC following in 2021 (Garber, 2022; Zhao et al., 2022). The TPD focuses on improved delivery of a pathogenic protein of interest (POI) to the degradation machinery. This is achieved by the formation of a stable delivery ternary complex that is tagged with ubiquitin. An E3-recruiting ligand with ubiquitin tags bind to the PROTAC protein, and the PROTAC binds to the POI (Sun et al., 2019; Wang et al., 2020; Yang et al., 2021; Zhao et al., 2022). The ubiquitin-tagged ternary complex is then delivered and degraded via the ubiquitin-proteasome system. The LYTAC, AbTAC, and molecular glue mechanisms are similar to PROTAC in that they deliver the POI to the degradation machinery but differ in where the POI is present. LYTAC targets extracellular POI by binding in the extracellular matrix and endocytosed into the cell. The ingested ternary LYTAC complex is enveloped into the early endosome, that later matures into the lysosome where the POI is degraded. AbTAC targets extracellular and membrane proteins for degradation in the lysosome. A bispecific, recombinant chimeric antibody is produced, with one arm targeting the POI and the other arm targeting a transmembrane E3 ligase, the most common being RNF43 (Zhao et al., 2022). The AbTAC complex induces internalization of the POI-complex and delivers the protein to the lysosome. Molecular glue molecules facilitate and increase the dimerization or colocalization of two proteins via forming a stable ternary complex (Gerry and Schreiber, 2020; Zhao et al., 2022), without the need to introduce a PROTAC. The first molecular glues discovered were the immunosuppressants cyclosporin A and FK506 (Schreiber, 1991) that induced the creation of stable ternary protein complexes. Application of TPD has been indicated in the treatments of cancer, inflammatory diseases, and viral infections, but its major implication is in neurodegenerative diseases (Zhao et al., 2022), specifically PD. Many studies are currently focused on development of PROTACs that target *α*-syn aggregates, LRRK2, and tau proteins (Amirian et al., 2023), all of which are implicated in the propagation and pathological symptoms seen in PD.

### Inflammatory machinery

During recent years it has been made clear that inflammation, specifically neuroinflammation, plays a major role in the dysfunctional environment and propagation of disease in neurodegenerative diseases (Glass et al., 2010; Chitnis and Weiner, 2017; Walker, 2018). The roles of adaptive versus innate immune responses shift during different stages of neurodegeneration, and these dynamics drive disease progression but also offer a therapeutic target to treat PD (Amor et al., 2010; Chitnis and Weiner, 2017). A major component of the immune system implicated as therapeutic targets in PD and other neurodegenerative diseases is a class of molecules labeled galectins.

Galectins are a family of carbohydrate (glycan) binding proteins that play an active role in inflammatory responses, mediation of cell–cell interactions, cellular differentiation, activation of macrophages and microglia, and apoptosis (Hara et al., 2020; Hisrich et al., 2020; Ramos-Martinez et al., 2022; Liu and Stowell, 2023). There have been 15 mammalian galectin proteins identified, and they all share a structure containing 130 amino acids and a carbohydrate recognition domain (CRD) region that recognizes ß-D-galactopyranoside glycan-binding (Thiemann and Baum, 2016; Johannes et al., 2018). Certain immune responses to galectins depend on the generation of specific glycosylation of membrane surface glycoproteins in order to generate galectin ligands.

Galectins are expressed across a multitude of cell and tissue types, and can be expressed constitutively or induced via a range of circumstances and stimuli, particularly in immune cells (Rabinovich et al., 2007; Martin-Saldana et al., 2022). Within cells themselves, a majority of galectins can be found within different cellular compartments. Galectin 3 (Gal-3) for example is found across the cell, including the cellular membrane where it modulates cell attachments, within the cytosol where it inhibits the cell’s intrinsic apoptosis pathway, and in the nucleus where it plays a role in gene transcription regulation (Kim and Chun, 2020; Martin-Saldana et al., 2022).

Due to their role in inflammation, patients diagnosed with neurodegenerative diseases experience fluctuating levels of galectins within their blood serum and brain tissues (Ramos-Martinez et al., 2022). While there is a vast number of galectins that play a role in the various neurodegenerative diseases, in PD specifically there are three galectins that play a major role: gal-1, gal-3, and gal-4 (Martin-Saldana et al., 2022). Gal-1 and gal-3 are the two most studied galectins, and their roles are well characterized in innate and adaptive immune responses (Martin-Saldana et al., 2022). Gal-1 is a prototype galectin with a single CRD and is located within the extracellular matrix (ECM), while gal-3 is a chimeric galectin with a single CRD and an additional tandem repeat sequence that allows oligomerization after binding to a ligand within the ECM (Martin-Saldana et al., 2022). Gal-4, while not as well understood, still plays a major role in PD. Gal-4 is a tandem repeat galectin that has two CRDs in tandem and is associated with the cellular membrane (Martin-Saldana et al., 2022).

Gal-1 has been located within neurons, astrocytes, microglia, and the cerebrospinal fluid (CSF) of PD patients (Martin-Saldana et al., 2022). Gal-1 has been implicated in the process of neurogenesis regulation, oligodendrocyte maturation, and myeline development, as well as direct activation of microglia, suppression of microglia, and anti-inflammatory cascades via modulation of the MAPK/IκB/NFκB axis (Li et al., 2020). Gal-1 was detailed to ameliorate microglial activation and provide a neuroprotective ability to prevent neuron degeneration not only *in vitro* but also *in vivo* (Li et al., 2020). Gal-1 is able to exert this function on the MAPK/IκB/NFκB axis via its CRD. In a more recent study, gal-1 was also able to exert neuroprotective effects via regulation of nuclear factor erythroid 2-related factor (NRF2) expression (Liu et al., 2022). Due to its recently detailed neuroprotective abilities, gal-1 still needs more research but is an illustrious target for PD treatment.

Gal-3 has been located within microglia and the blood serum of PD patients (Martin-Saldana et al., 2022). The exact role gal-3 plays in PD is not yet completely understood, but studies have shown it has a detrimental effect in PD and neurodegenerative diseases (Garcia-Revilla et al., 2023). Increases in gal-3 leads to the activation of microglia and promotion of chronic neuroinflammation which leads to an increase in neurodegeneration. Cells that are burdened with *α*-syn form gal-3 puncta due to an increase of ROS and leads to vesicle rupture downstream (Garcia-Revilla et al., 2017). The increase in vesicle rupturing releases enclosed molecules that are introduced into the cytoplasm and can increase the mitochondrial dysfunction within the cell. The measured levels of gal-3 continuously increase as the progression of PD continuously worsens, and may be able to be utilized as a disease stage tracking mechanism (Cengiz et al., 2019; Martin-Saldana et al., 2022).

Gal-4 is not as well characterized or studied as its gal-1 and gal-3 counterparts. Gal-4 has been located in neurons, oligodendrocytes, and within the blood serum of PD patients (Martin-Saldana et al., 2022). Gal-4 has been implicated in the growth of axons and suppression of the myelination that surrounds them. It has also been noted that like gal-3, the levels of gal-4 increase in proportion with the progression of PD (Cengiz et al., 2019), making gal-4 a possible biomarker for PD diagnosis and stage progression.

The cellular machinery utilized in PD research is summarized in Table 1. Cell therapy options and research is detailed in Table 2.

**Table 1:** Cellular mechanisms.

	Class	Therapeutic	Mechanism	Target	Disease	Utilization
Degradation Machinery	Targeted Protein Degradation (TPD)	PROTAC	An E3-recruiting ligand tagged with ubiquitin binds to the TPD machinery that is attached to the protein of interest and efficiently delivers it to the proteosome for degradation	*α*-syn, Tau	PD	Targets intracellular proteins of interest and efficiently delivers them to be degraded
		LYTAC		*α*-syn	PD	Targets extracellular proteins of interest and is endocytosed into the cell
		AbTAC		*α*-syn	PD	Targets membrane and extracellular proteins of interest and delivers to the lysosome
		Molecular Glue		*α*-syn	PD	Directly facilitates dimerization of protein of interest and the ubiquitin-proteosome without the addition of a PROTAC
	Protein	ATPase valosin-containing protein (VCP)	ATP-dependent unfolding of proteins via ubiquitin-proteasome pathway	Tau	AD/PD	Strong activity *in vitro* but not as efficient *in vivo*, but is still a therapeutic target for aggregate clearance
	Drug	Aryloxy propanolamine	Chemical dissociation of ß-sheets in Aß aggregates	Aß	AD	Alleviates pathological symptoms and aggregate burden in mice models
Inflammatory machinery	Galectins	Gal-1	Family of native glycan binding proteins that play an active role in both the adaptive and innate immune systems, regulation of cellular contacts, and other cellular behaviors	MAPK/IκB/NFκB axis	ALS/MS/PD	Binding and interaction with the MAPK/IκB/NFκB axis exerts neuroprotective effects, and upregulation holds promise as a PD therapeutic
		Gal-3		*α*-syn	AD/ALS/PD	Downregulation and knockout models have proven to reduce PD progression and exerts a neuroprotective effect
		Gal-4		Not well characterized in PD	PD	Levels of gal-4 increase as PD progresses and may be used as a biomarker for diagnoses, further studies into gal-4 inhibition are needed

**Table 2:** Cell therapy.

	Normal physiological role	Target	Therapies
Microglia	Brain resident macrophages that provide primary immune defense, maintenance of neuronal homeostasis, and degradation of pathogenic proteins and materials	Enhancing phagocytosis, reducing neuroinflammation, and genetic engineering to perform necessary functions more efficiently all increase the neuroprotective potential of microglia for DA neurons	Naloxone, celecoxib, bioengineering CRISPR, ginsenoside Rg1, piperine, rosmarinic acid, astilbin, curcumin
Astrocytes	Specialized glial cells that perform supportive roles for neurons by regulation of trophic factors and oxidative stress, structural scaffolding, metabolic homeostasis, and neuroprotection	Regulation of astrogliosis, replacement of dysfunctional astrocytes, and reduction of chronic astrocytic inflammation	Regulators and inhibitors of the MAPK, NF- κB, calcineurin, and JAK/STAT pathways
Microglia-astrocyte cross talk	Two supportive and neuroprotective cell types maintain constant communication in order to increase overall ability to perform vital functions and more efficiently degrade toxic protein aggregates	The ability of communication between microglia and astrocytes has been shown to increase both cell types’ ability to degrade pathogenic *α*-syn aggregates	Understudied, but increasing communication between supportive cells is a promising treatment for PD
Natural killer (NK) cells	Innate immune cells that provide first line defense against pathogens, viruses, microbes and malignant cells via scavenging the brain	Increasing NKCC and secretion of IFN- *γ* to activate NK cells leading to an increase of scavenging of *α*-syn aggregates and clearances of aged and diseased cells	Understudied, but increased targeting of *α*-syn aggregates is a promising treatment for PD
Chimeric antigen receptor (CAR)	Bioengineered; no normal physiological role	Receptor protein can be engineered to target any specific protein (i.e. *α*-syn, Aß, Tau) or antibody that attaches to pathogenic protein aggregates	Bioengineering, CRISPR, antibody pairing

### Cell therapy

The human brain is comprised of a complex network of a multitude of cell types, all functioning together to perform all the necessary, vital operations. These cell types include neurons, glial cells, and immune cells. Upon being structured in a highly ordered matrix, these cells interact in a delicate balance in order to facilitate and execute every activity of the human brain.

Neurons are the fundamental units that comprise the brain and nervous system. Neurons relay important information through electrical impulses and neurotransmitters in order to carry out a specific function. Neurons represent an extraordinarily diverse class of cell types that differ across molecular, morphological, physiological, and connective features (Peng et al., 2021), as well as the specific location in the brain. Neurons with similar physiological objectives connect and form neural pathways, a network of neurons that work and communicate together to accomplish a specialized job. Neural networks and individual neurons require a support network in order to correctly function. These supports are comprised of glial cells, a large class of brain cells that were originally defined as “nonneuronal cells with support functions” (Araque and Navarrete, 2010). Glial cells were first described in detail in the 19^th^ century by neuroscientist Rudolf Virchow, who labeled glial cells with the German word “*nervenkitt*,” translated as “nerve glue” in English (Jakel and Dimou, 2017). Traditionally, glial cells were thought to only provide support for neurons, but more recent studies have shown that glial cells have a vastly wider array of functions (Garcia-Bermudez et al., 2021), including generation of axonal myelin sheaths, production and protection of synapses, and maintenance of neurotransmitters at physiological levels (Uddin and Lim, 2022). In order to maintain a functioning, healthy environment in which neurons and glial cells can operate, immune cells patrol the brain in order to modulate inflammatory signals (Norris and Kipnis, 2019), induce phagocytosis and clearance of debris, dead cells (Ousman and Kubes, 2012), and protein aggregates (Lim and Yue, 2015), and respond to invading pathogens (Schwartz et al., 2022) and microbes (Nevalainen et al., 2022). Recent advancements in the field have increasingly turned their focus to utilizing resident neuronal cells to better understand the encompassing effects of PD pathology in all parts of the brain, the biochemical and biophysical interactions of varying cell types in PD, and to attempt to alleviate PD symptoms via elimination of *α*-syn aggregate burden in cells.

### Microglia

Microglia are resident macrophages in the brain and have a multitude of integral functions as they are involved in neural development, cognitive functions, and immune responses (Luo and Sugimura, 2024). Microglia are responsible for synapse pruning, injury repair, and maintaining homeostasis within the central nervous system (CNS) as well (Xu et al., 2021). Microglia are a diverse classification of cells because they display extensive phenotypic variability due to their sensitivity to stimuli and changes in the CNS microenvironment and perform necessary functions under both normal and abnormal conditions (Zhu et al., 2022). Microglia have the innate ability to ingest and degrade *α*-syn aggregates and attenuate their neurotoxic potential (Chen and Colonna, 2021). This mechanism, however, is not perfect and is accompanied with a major cost. At higher rates of accumulation of these toxic aggregates, the microglia become overwhelmed with alpha synuclein, resulting in endoplasmic reticulum stress, increased mitochondrial dysfunction, and increased release of proinflammatory cytokines (Chen and Colonna, 2021; Scheiblich et al., 2021). When homeostasis is disrupted, microglia function affects the progress neurodegenerative diseases, including PD (Xu et al., 2021; Shao et al., 2022). Potential therapeutic targets for microglia include enhancing phagocytosis to clear pathogenic aggregates, reducing microglial-induced neuroinflammation to protect sensitive neurons, inhibiting microglial creation and secretion of exosomes containing harmful protein aggregates, transformation of microglia into a neuroprotective phenotype, and genetic engineering microglial cells to perform necessary functions at a higher propensity (Gao et al., 2023).

Modulating microglial activation and their influence on neuroinflammation has been of key interest. Commercial drugs, like naloxone and celecoxib, have shown abilities to protect DA neurons, albeit via separate mechanisms (Gao et al., 2023). Naloxone, an opioid antagonist, binds to opioid receptors in the brain. In multiple published studies, naloxone has proven to decrease microglial activation and limit neuroinflammation by reducing lipopolysaccharide (LPS) induced production of cytokines, block morphological changes of microglia into an activated state, and inhibit generation of superoxide free radicals by the microglia (Liu et al., 2000; Lu et al., 2000; Wang et al., 2012). Celecoxib, a nonsteroidal anti-inflammatory drug (NSAID), has been shown to selectively inhibit COX-2, the inducible gene responsible for production of prostaglandins (Simon, 1999), and prevent progressive dopaminergic neuron degeneration in the brains of a rat PD model (Sanchez-Pernaute et al., 2004). Rather than pharmaceutical options, scientists have also studied the effect of natural compounds on neuroinflammation and microglia inactivation, including ginsenoside Rg1 (Heng et al., 2016), piperine (Yang et al., 2015), rosmarinic acid (Lv et al., 2019), astilbin (Zhu et al., 2019), and curcumin (Ghasemi et al., 2019). Curcumin, the bright yellow active compound in turmeric, has also been shown to play an active role in mitochondrial biogenesis, another aspect that is being considered in the treatment of neurodegenerative diseases and PD (Hamidie et al., 2021; Sathyabhama et al., 2022).

Engineering microglial cells can be achieved through many differing genetic mechanisms, including insertions, deletions, or CRISPR-Cas9 editing of target genes. Insertion of beneficial genes via viral transduction offers a viable path at controlling and improving the microglial function in the patient, while deletion of pathogenic genes promotes polarization toward neuroprotective states (Luo and Sugimura, 2024).

In a recent study, Plasschaert et al. (2022) developed and characterized genetically modified microglia-like cells (MLCs) as a potential gene therapy for neurodegenerative diseases. The fabrication of the genetically modified MLCs consists of extracting hematopoietic stem/progenitor cells (HSPC) from the patient, the transduction with a lentiviral vector carrying a therapeutic payload, and intravenous reintroduction of cells back into the patient (Plasschaert et al., 2022). HSPC gene therapy (HSPC-GT) is of particular interest in the treatment of PD due to the cells’ ability to cross the blood–brain barrier after intravenous administration (Biffi et al., 2006; Plasschaert et al., 2022). The group showed that MLCs administered via simple intravenous injection resulted in “widespread engraftment of genetically engineered cells” throughout the periphery of the brain and improved the immune function of the cells (Plasschaert et al., 2022).

### Astrocytes

Astrocytes are a specialized subtype of glial cells that constitute the majority of cells within the human brain, outnumbering neurons between 2:1 and 3:1 (Fang et al., 2019). Astrocytes perform a multitude of functions in order to support the correct functioning of neurons, including metabolic, structural, homeostatic, neuroprotective roles, blood–brain barrier maintenance and stabilization, and promotion of synaptic formations (Vasile et al., 2017). Astrocytes also play an active role in releasing beneficial trophic factors, regulation of oxidative stress via production of antioxidants, and endocytosing and degrading toxic molecules from the extracellular matrix (Man et al., 2018). Their supportive functions are vital for the creation of a nurturing environment for neurons within the brain, and to ensure this environment is maintained, astrocytes are in constant communication with neurons via gap junctions, ion channels, foot processes (Benarroch, 2005), and tunneling nanotubes (Khattar et al., 2022).

Research focusing on astrocytes as a potential therapeutic in neurodegenerative diseases has turned to astrogliosis. Astrogliosis is the state in which astrocytes activate a coordinated response to an array of abnormal signals, including central nervous system injury, disease progression, overproduction of reactive oxygen or nitrogen species (ROS/RNS), or dysfunction in the detoxification mechanisms (Rizor et al., 2019). Activated astrogliosis is a beneficial protective mechanism in short term periods, especially during early etiology of disease, but chronic astrogliosis leads to sustained release of proinflammatory signals. Prolonged exposure to inflammatory cytokines propagates the loss of dopaminergic neurons and worsens PD pathology (Rizor et al., 2019). Scientists in the field are looking at potential mechanisms to replace dysfunctional astrocytes, discover a pharmacological treatment that targets the detrimental chronic activation of astrogliosis, and convert activated astrocytes back into a neuroprotective state.

Replacement of dysfunctional astrocytes in order to alleviate human disease is not a new concept in science. However, the premise of astrocytic replacement therapy has garnered new interest in neurodegenerative diseases. In one of the first studies of astrocytic replacement, it was discovered that transplantation of lineage-restricted astrocytes precursors led to the cells being able to survive within a diseased tissue and differentiate into beneficial astrocytes (Lepore et al., 2008). The therapy extended life expectancy in the dysfunctional astrocyte transgenic mice used in the study, while also attenuating motor neuron loss within the brain (Lepore et al., 2008). Another study confirms that the introduction of glial restricted precursor cells (GRPCs) via transplantation are shown to increase the release of neuroprotective trophic factors, increase the release of mediative antioxidants, improve behavioral deficits in PD mice, and reinstate healthy levels of the enzyme tyrosine hydrogenase (TH), which plays an important role in the biosynthesis of dopamine (Proschel et al., 2014; Man et al., 2018).

Another aspect of astrocyte therapies focuses on direct targeting of inflammation signaling to reduce chronic neuroinflammation. Within the biochemical environment of the brain, there are multiple pathways in which astrocytes are modulated between normal and astrogliosis states (Giovannoni and Quintana, 2020). The Janus kinase/signal transducer and activator of transcription 3 (JAK/STAT3) pathway is responsible for the initiation of astrogliosis (Herrmann et al., 2008; Ceyzeriat et al., 2016), and activation of this pathway is a hallmark feature of reactive astrocytes in many animal models of PD and other neurodegenerative diseases (Ben Haim et al., 2015). Contrarily, the nuclear factor kappa light-chain-enhancer of activated B cells (NF-κB) pathway (Brambilla et al., 2005), the calcineurin pathway (Furman and Norris, 2014), and the mitogen-activated protein kinase (MAPK) pathway (Roy Choudhury et al., 2014) all play an active role in mediating astrocytic reactivity towards a normal state (Giovannoni and Quintana, 2020). These biological pathways exist in a delicate balance in the healthy brain environment, but drastically alter disease progression when that balance is disturbed.

The JAK/STAT pathway is the overarching predominant pathway used by cytokines in their functionality to modulate inflammatory responses and the innate immune system, with over 70 cytokines utilizing the pathway (O'Shea et al., 2015; Qin et al., 2016). In a particular study, scientists determined that overexpression of *α*-syn in dopaminergic neurons activates the JAK/STAT pathway, leading to a dysfunction in the immune response and ultimately neurodegeneration in a rat model (Qin et al., 2016). This group treated the alpha-synuclein overexpressing rats with a JAK inhibitor (AZD1480). Treatments proved to have a therapeutic effect via suppression of the JAK/STAT pathway, thus reducing neuroinflammation and the degeneration of dopaminergic neurons in the PD rat model (Qin et al., 2016). AZD1480 has also shown the ability to reduce microgliosis and macrophage infiltration, and reduce the secretion of proinflammatory cytokines (Lashgari et al., 2021). While JAK inhibitors have been approved by the US Food and Drug Administration (FDA) for certain usages, it should be noted that the FDA placed a black box warning on the drug in 2019, and in previous clinical studies certain JAK inhibitors have raised safety concerns over impacts on hematopoiesis (Lashgari et al., 2021), drastic immunosuppression of the patient, and venous thromboembolism (Samuel et al., 2023). Nonetheless, several research groups continue investigating the potential of the JAK/STAT system in PD treatment.

### Microglia-astrocyte cross talk

Microglia and astrocytes are both vitally important to the overall health and functioning of the brain in their own respects, but studies have shown that the interaction between these two cell types has profound effects. In a 2021 study conducted by Rostami et al. (2021), it was shown that astrocytes accumulate *α*-syn fibrils at a much higher rate than microglia. Not only did the group show the uptake of alpha synuclein, but the data presented showed that the astrocytes were able to degrade a portion of the aggregates as well. The endocytosed protein aggregates enter the endo-lysosomal pathway and are subsequently degraded (Rostami et al., 2020), although the microglia in the study showed a more efficient ability to degrade the *α*-syn over the astrocytes (Rostami et al., 2021).

When astrocytes and microglia were in a co-culturing environment, the overall levels of *α*-syn intracellularly and extracellularly were decreased at the 7-day time point (Rostami et al., 2021). Astrocytes and microglia are in constant communication with each other via multiple mechanisms. The group of scientists observed that the astrocytes in the experiment preferentially endocytosed the free *α*-syn that was then transferred to the microglia via secretory exosomes. The cell types also exhibited extensive connections via tunneling nanotubes, with the ability of the astrocytes to transport aggregates to the microglia via this path. This vital crosstalk interaction between astrocytes and microglia in brain tissue could possibly play a major role in PD treatments and therapeutics in the future.

### Natural killer cells

Amongst the other cell types already discussed, brain tissue also contains natural killer (NK) cells. NK cells are large innate immune cells that are classified in the group 1 innate lymphocyte (ILC1) family (Brauning et al., 2022). NK cells act as scavengers and are a first line of defense against viruses, intracellular pathogens, microbes, or malignant cells (Vivier et al., 2008; Brauning et al., 2022), with a more recent discovery in the clearance of senescent cells (Antonangeli et al., 2019; Song et al., 2020). NK cells play this active defensive role via elimination of targets through direct cell-to-cell contact. Due to their important role in the immune process, as well as aging, NK cells have been a major target in PD therapeutics.

In a recent study, Earls et al. (2020) investigated the effect of NK cells on the severity of PD pathology in a preformed fibril (PFF) mouse model. Within the study, the scientists concluded that NK cells are able to effectively clear the aggregated *α*-syn without aberrant activation. Due to chronic inflammation seen in PD, the blood–brain barrier becomes disturbed and allows for non-resident immune cells to cross into the brain (Garretti et al., 2019). Once inside the brain tissues, NK cells are available to scavenge and degrade free extracellular aggregates of *α*-syn via the endo-lysosomal pathway. Upon depletion of NK cells within the system, the mice exhibited a significantly exacerbated PD pathology with increased motor deficits compared to the control counterparts, and brain tests exhibited an increased occurrence of phosphorylated *α*-syn deposits (Earls et al., 2020). This suggests that NK cells play a pivotal role in protection of dopaminergic neurons in neurodegenerative diseases.

Earls et al. also showed that *α*-syn reduces NK cell cytotoxicity (NKCC) in a dose-dependent relationship and decreases the secretion of IFN- *γ*, a proinflammatory cytokine.

Multiple studies have concluded that the population of NK cells changes not only within locations of the nervous system, but also changes during aging and the progression of PD pathology (Earls et al., 2020; Menees and Lee, 2022). Amidst the aging process in humans, the immune system undergoes a significant change. The overall population of NK cells increase and experience a redistribution of phenotypic and functionality (Hazeldine and Lord, 2013; Solana et al., 2018). As PD propagates and the neurodegeneration worsens, NK cells cluster in higher percentages in the patient’s blood and in effected areas of the brain (Menees and Lee, 2022). During postmortem autopsies of PD patient brain samples, scientists have observed high incidences of NK cells colocalized in areas robust in phosphorylated *α*-syn inclusion bodies (Earls et al., 2020) via utilization of immunohistochemical analysis. Historically, the role of NK cells had been understudied, but their ability to act as *α*-syn scavengers, clear aged and diseased cells (Earls and Lee, 2020), and act as immune cells have opened the door for the field to focus on NK cells as a potential therapeutic target for PD treatment.

### Chimeric antigen receptor engineered cells

**Figure 5:**
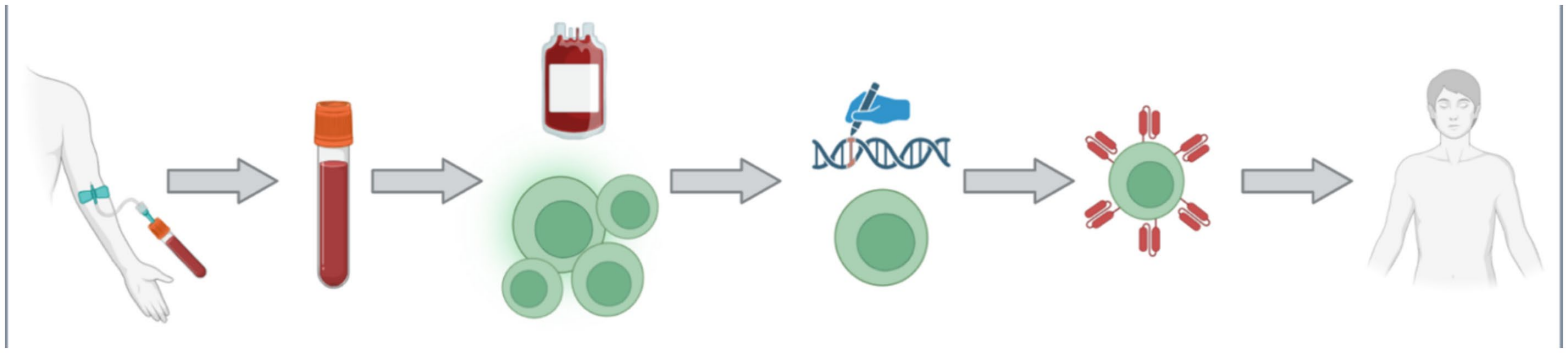
The process of CAR T-cell is multifaceted with multiple steps. Firstly, a patient donates their blood via an intravenous collection method. The collected blood is treated such that the specific patient’s T-cells can be collected, and the rest of the blood may be returned back into the patient. The collected T-cell are then transported to the laboratory for genetic modification. The introduced genetic modifications allow the cells to produce and manifest the specific CAR protein on their membrane. The newly bioengineered T-cells are then returned into the patient as an increasingly efficient therapy. Figure was created using BioRender.com.

The largest expanse of promise in PD research within the last half decade has been the field of chimeric antigen receptor (CAR) engineered immune cells. CAR immunotherapy technology is not new to science, as it was first characterized in the late 1980s (Kuwana et al., 1987; Dotti et al., 2014). This technology is most renowned for its application in cancer treatment, where CAR T-cell therapy remains the leading utilization. An individual cancer patient’s blood is collected, and their T-cells are harvested. The resulting blood is then returned into the patient’s circulation, while the T-cells are delivered to a laboratory for transformation. The T-cells are bioengineered to present a CAR that is specific to the patient’s cancer cells in order for the improved immune cells to act more efficiently in cancer treatment (Levine et al., 2017). The multistep process of CAR T-cell therapy is depicted in Figure 5, from the clinic to the laboratory and back to the clinic.

More recently, scientists have been applying CAR technology to other cell types rather than the tradition T cells. Since NK cells play an active role in the immune system as first responders, researcher have created CAR-NK cell therapies in the immunotherapy cancer treatment setting. In the presence of cancer cells expressing foreign, “non-self” proteins, NK cells are activated and eliminate the cancer cell via its cytotoxic mechanism (NKCC) and concurrently secrete proinflammatory cytokines in order to activate the other aspects of the immune system (Zhang et al., 2024). The expression of the CAR protein focuses the NK cells’ potential and specificity of its immune response, thus increasing the efficiency as a therapeutic. CAR T lymphocytes can only target and kill cells that express the specific target protein of the carried CAR, while CAR-NK cells can independently enact their cytotoxicity and use the carried CAR protein to recognize malignant cells (Habib et al., 2019). Therefore, CAR-NK therapy is already an attractive method of immunotherapy in medicine due to its ability to be fine-tuned and prevent immune-mediated adverse events (AEs) (Habib et al., 2019), and potentially has profound effects on the neurodegeneration field if a successful CAR is produced that recognizes pathogenic aggregates.

The premise of using CAR technology is so promising that researchers have already begun studying CAR T cells in areas beyond cancer, such as in neurodegenerative diseases like Alzheimer’s disease (AD) (Sarko and Saha, 2023) and multiple sclerosis (MS) (Mullard, 2024), autoimmune diseases like lupus and myasthenia gravis (Mullard, 2024), and even allergies and infectious diseases as well (Zmievskaya et al., 2021). In each separate application, the CAR protein is specially engineered to accomplish its therapeutic task, rather it is to recognize a malignant protein, a viral surface protein, or pathogenic components. The pivotal step for using CAR as a therapeutic in PD is identifying an appropriate target to engineer the CAR to recognize.

There are many potential targets to aid in the treatment of PD, but two promising mechanisms hold great promise: targeting exosome surface proteins and targeting an anti-*α*-syn antibody. It has been well studied and documented that exosomes carrying aggregated pathogenic *α*-syn contribute to the propagation of PD (Yu et al., 2020; Pinnell et al., 2021; Ouerdane et al., 2022), and is one of the three major mechanisms of aggregate spreading in the brain, as depicted previously in Figure 3. Exosomes are membrane bound extracellular vesicles produced by all cells as a mean of positive communication with surrounding cells carrying information molecules, important cellular proteins, DNA and RNA, metabolites, and lipids, but also as a means of discarding and purging toxic materials and protein aggregates (Kalluri and LeBleu, 2020). Exosomes exhibit a series of exosome-specific surface proteins that belong to the tetraspanin super family (Ivanusic and Denner, 2023). Scientists have routinely been using these surface proteins as a means to purify, differentiate, and target exosomes, specifically using CD9, CD63, and CD81 proteins (Escola et al., 1998; Fordjour et al., 2022; Ivanusic and Denner, 2023). In the cancer setting, researchers have already created and tested CAR T cells that target CD positive exosomes and have shown a positive uptake in those exosomes carrying tumorigenesis antigens and factors (Ukrainskaya et al., 2023). An intriguing prospect in the therapeutic use of this technology in PD is engineering a CD-CAR protein and expressing in cells that have previously shown activity in clearing *α*-syn aggregates, like NK cells and microglia.

Another potentially impactful field of CAR therapeutics in PD revolves around the use of antibodies. Antibodies are naturally produced proteins by the cells in the immune system as a defense mechanism against a range of foreign molecules and pathogens (Hummer et al., 2022). Utilizing antibodies against *α*-syn in PD poses significant health risks that need to be considered. Alpha synuclein, although its exact physiological role has not been detailed, is still a functioning protein used by cells. If an antibody is improperly binding with all forms of alpha synuclein, there are on-target deleterious effects (Henderson et al., 2020). Antibodies freely navigate the body via the circulatory system and also cross the blood–brain barrier via receptor-mediated transport (Neves et al., 2016). Due to levels of *α*-syn in the red blood cells (Scherzer et al., 2008), careless introduction of antibodies that bind non-specifically to all types of *α*-syn can lead to detrimental effects. Therefore, a meticulous design of antibodies that target only misfolded and aggregated forms of *α*-syn has immense therapeutic potential. Studies have already been conducted that produced highly conformationally specific antibodies against pathogenic forms of *α*-syn capable of reducing *α*-syn pathology in multiple regions of the mouse brain (Henderson et al., 2020). A previous study conducted by Vaikath et al. (2015) reconfirms that the production of conformationally-specific anti-*α*-syn antibodies portrayed as useful tools for further research, biomarkers development, diagnostics, and as an immunotherapy for PD. In a more recent study, Altay et al. (2023) studied a wide range of potential anti-*α*-syn antibodies based of modifications to the *α*-syn protein itself, including phosphorylation, truncation, and nitration that results in a pathogenic protein.

Pairing the technology of pathogenically specific antibodies with CAR engineered cells in order to clear *α*-syn aggregates in the brains of PD patients is a very attractive therapeutic strategy. Similar mechanisms of utilizing the antibody-CAR cell interaction have already been reported in other diseases. Stock et al. (2022) discovered that this mechanism is useful in the treatment of solid tumors, like HER2- breast cancer. Antibody-CAR cell therapy has even been introduced into a phase 1 clinical trial in the treatment of gastric and pancreatic cancers (Kalluri and LeBleu, 2020). The enhanced efficiency of antibody-CAR engineered cell therapy opens the door to a new wave of research across a variety of diseases, but has an especially interesting application in the treatment of neurodegenerative diseases and PD.

## Conclusion

Over the past decade, major progress in the understanding of the underlying molecular cause of PD was made. Microscopic analysis of LBs, intracellular formations observed in midbrain, hypothalamus and thalamus of PD patients, revealed the presence of *α*-syn fibrils and fragments of cell membranes. Biophysical and biochemical methods confirmed that such fibrils are highly cytotoxic to neurons, whereas lipids could drastically alter their secondary structure and toxicity. These and other pieces of experimental evidence indicated that the onset of PD was caused by the aggregation *α*-syn, while the spread of this pathology in the brain was linked to *α*-syn oligomers and fibrils. At the same time, numerous attempts to discover low molecular weight compounds that could inhibit such aggregation were largely unsuccessful. This triggered an interest in alternative strategies that can be used to treat PD. In this review, we critically discussed the most recent studies that employed chimeric antigen receptor and immune cells to degrade *α*-syn fibrils. We review advantages and disadvantages of these novel therapeutic approaches and demonstrated the extent to which cell therapy could be used to treat PD.

## Author contributions

CM: Conceptualization, Visualization, Writing – original draft, Writing – review & editing. DK: Funding acquisition, Project administration, Resources, Supervision, Writing – review & editing.
